# Severe COVID-19: understanding the role of immunity, endothelium, and coagulation in clinical practice

**DOI:** 10.1590/1677-5449.200131

**Published:** 2020-11-16

**Authors:** Simone Cristina Soares Brandão, Emmanuelle Tenório Albuquerque Madruga Godoi, Júlia de Oliveira Xavier Ramos, Leila Maria Magalhães Pessoa de Melo, Emanuel Sávio Cavalcanti Sarinho

**Affiliations:** 1 Universidade Federal de Pernambuco – UFPE, Recife, PE, Brasil.; 2 Serviço de Hematologia de São José dos Campos, São José dos Campos, SP, Brasil.

**Keywords:** COVID-19, endothelium, immunity, atherosclerosis, thrombosis

## Abstract

SARS-CoV-2 is responsible for the COVID-19 pandemic. The immune system is a determinant factor in defense against viral infections. Thus, when it acts in a balanced and effective manner the disease is self-limited and benign. Nevertheless, in a significant proportion of the population, the immune response is exaggerated. When infected, patients with diabetes, hypertension, obesity, and cardiovascular disease are more likely to progress to severe forms. These diseases are related to chronic inflammation and endothelial dysfunction. Toll-like receptors are expressed on immune cells and play an important role in the physiopathology of cardiovascular and metabolic diseases. When activated, they can induce release of inflammatory cytokines. Hypercoagulability, hyperinflammation, platelet hyperresponsiveness, and endothelial dysfunction occur in immune system hyperactivity caused by viral activity, thereby increasing the risk of arterial and venous thrombosis. We discuss the interactions between COVID-19, immunity, the endothelium, and coagulation, as well as why cardiometabolic diseases have a negative impact on COVID-19 prognosis.

## INTRODUCTION

The severe acute respiratory syndrome coronavirus 2 (SARS-CoV-2) is responsible for COVID-19 disease (COronaVIrus Disease 2019). Coronaviruses cause outbreaks that threaten health, including the 2002 severe acute respiratory syndrome (SARS) epidemic in China, the 2012 Middle East respiratory syndrome (MERS), and the current COVID-19 pandemic.^1^^-^3

SARS-CoV-2 was identified in patients with atypical pneumonia, characterized by fever, dry cough, and progressive dyspnea.^1^^,^^4^^-^6 The initial data from China, showed that 81% of COVID-19 cases exhibited mild to moderate symptoms, including patients with no pneumonia or with mild pneumonia. Fourteen percent of patients developed the severe disease and 5% became critically ill, with respiratory failure and multiple organ failure and an extremely high risk of death.^1^^,^^7^^-^9

The major question is why some people progress to the severe form of COVID-19. Studies have shown a significant relationship between disease severity and immune system markers. It was suggested that during the response to SARS-CoV-2, immunological dysregulation and elevated levels of proinflammatory cytokines could be the primary cause of tissue damage. More than anything, it is people with cardiovascular diseases (CVD) and metabolic diseases who are at the highest risk of death from COVID-19.^2^^,^^10^^-^12

Therefore, this pandemic has created many challenges for medicine, since there is an urgent need to save lives. We know that immunresponse, the endothelium, and the coagulation system interact intimately and that chronic inflammatory processes are involved in the pathophysiology of CVD and metabolic diseases. This review discusses the interactions between COVID-19, immunity, the endothelium, and coagulation, and also the possible aspects that induce cardiometabolic diseases to have a negative impact on COVID-19 cases. The objective is to stimulate increased reflection on the influence of these factors on treatment approaches to this new disease.

## COVID-19, IMMUNORESPONSE AND DISEASE PHASES

The immune system is activated to defend against infectious agents. Initially, this function is mediated by innate immunity reactions and later by adaptive immunity reactions, which are determinant in combating viral infections. In COVID-19, an effective and balanced inflammatory response enables a self-limiting and benign disease course. The severe form occurs in a subset of patients whose immunoresponse to SARS-CoV-2 is exaggerated.^1^^,^^13^^-^15

SARS-CoV-2 is a member of the betacoronavirus family, it has single-stranded RNA with typical structural proteins, comprising envelope (E protein), membrane (M protein), nucleocapsid (N protein), and spike (S protein) proteins, the last of which is responsible for the virus’ infectiousness.^16^^,^^17^ The S proteins on the surface of SARS-CoV-2 bind to human ACE2 receptors (angiotensin-converting enzyme 2), transmembrane proteins that, in turn, transfer the genetic material into the cell and initiate the replication process.^16^^-^18

Human cells infected by the virus are recognized by the immune systems, both innate and adaptive, which triggers cytokine production. Two of the most important cytokines produced are tumor necrosis factor alpha (TNF-α) and interferon-gamma (IFN-γ). The first is responsible for activation of neutrophils and promotion of coagulation and acts on a centralized level to produce fever; the second induces macrophage activity to destroy the pathogen and amplifies the release of pro-inflammatory, pro-fibrotic, immunoresponse-regulating cytokines.^2^^,^^10^^,^^14^^,^^19^^-^21

**Figure 1:**
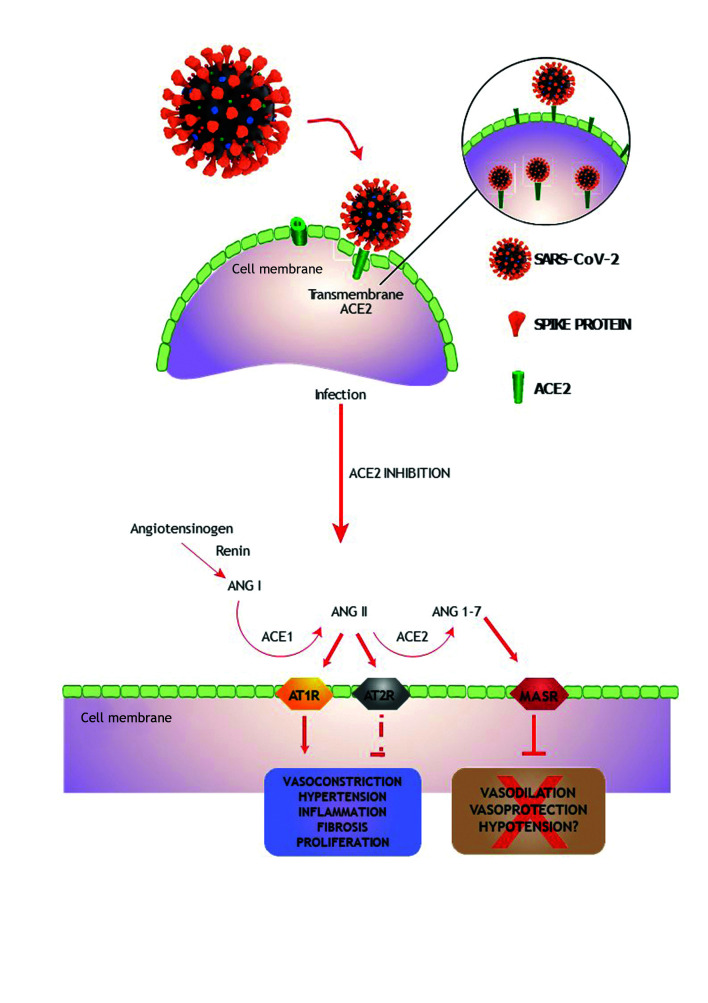
Activities of angiotensin (1-7), subproduct of angiotensin 1, and consequences of blocking. Angiotensin-converting enzyme 1 (ACE1) and angiotensin-converting enzyme 2 (ACE2) act on angiotensins 1 and 2. The SARS-CoV-2 (severe acute respiratory syndrome coronavirus 2) S protein binds to the ACE2 receptor of the human cell, reducing its enzyme activity. AT1R = type 1 angiotensin II receptor; AT2R = type 2 angiotensin II receptor; MASR = angiotensin 1-7 MAS receptor.

Additionally, ACE2 is a carboxypeptidase capable of converting angiotensin 2 into angiotensins 1-7. This enzyme is a homolog of ACE1, but it fulfills the role of a counterweight in the renin-angiotensin-aldosterone system (RAAS).^16^^,^^18^^,^^22^ When the virus binds to the ACE2 receptor, production of angiotensins 1-7 is reduced, which can trigger a series of cardiovascular and proinflammatory complications (Figure 1).^18^

The phases of COVID-19 appear to be linked to the intensity of the immunoresponse. When there is an adequate inflammatory response, patients do not progress beyond phase I and the infection is resolved. In cases in which the immunoresponse is exaggerated, the disease progresses to phases II and III.

The mild form (phase I) is generally characterized by fever, dry cough, and tiredness. Other manifestations include diarrhea, myalgia, headaches, odynophagia, anosmia, ageusia, and coryza. The severe form (phase II) is characterized by dyspnea, tachypnea, low oxygen saturation, and pulmonary infiltrate visible on chest X-rays or computed tomography. Critical cases (phase III) show signs of circulatory shock, respiratory failure, and multiple organ failure.^2^^,^^4^^,^^6^^,^^7^^,^^23^^-^25

A modulated immunoresponse against SARS-CoV-2 appears to be essential for the resolution of COVID-19. It comprises coordinated production of proinflammatory (IFN-γ and TNF-α) and anti-inflammatory cytokines (interleukin-10 [IL-10]), which, together with cellular activity and immunoglobulins (Ig), activate the maximum potential for combating the virus.^14^^,^^19^^,^^20^

It is very important for patient management to define the disease phase by careful history taking.^6^^,^^7^ A syndrome involving pulmonary and extrapulmonary inflammation, known as secondary hemophagocytic lymphohistiocytosis (sHLH), may be present in phase III. It is characterized by immunological hyperactivity due to inadequate elimination of macrophages activated by natural killer (NK) cells and cytotoxic T lymphocytes, resulting in excessive production of proinflammatory cytokines,^2^ followed by a cytokine “storm” and exacerbation of the inflammatory mechanisms. This condition can cause death.^2^^,^^25^

Therefore, patients with severe COVID-19should be screened for hyperinflammation, using laboratory markers and the HScore.^26^ This score was the first score to be validated for the diagnosis of reactive hemophagocytic syndrome. It is based on the largest dataset from adult patients with suspected reactive hemophagocytic syndrome and comprises nine appropriately weighted clinical, biological, and cytological variables. A cutoff of >169 on the HScore has 93% sensitivity and 86% specificity for sHLH.^26^

The intensity of the immunoresponse to SARS-CoV-2 interferes negatively with endothelial function and preexisting diseases related to endothelium are factors associated with the severity of COVID-19. This communication network makes it clear that, in the severe form, the immune system/hyperinflammation, the endothelium, and coagulation are involved cyclically.

## ENDOTHELIAL RESPONSE TO COVID-19

The endothelium is an active organ with endocrine, paracrine, and autocrine functions, and is indispensable for the regulation of tonus and maintenance of vascular homeostasis.^5^^,^^27^ In COVID-19, regardless of whether recruitment of immune cells it is immunomediated or in response to direct viral aggression of the endothelium, it can cause generalized endothelial dysfunction associated with apoptosis.^5^^,^^8^^,^^28^^-^30

In cases of greatest severity, death is caused by multiple organ failure. Post mortem histological studies have revealed a picture of lymphocytic endotheliitis in the lungs, heart, kidneys, and liver, and also cellular necrosis and presence of microthrombi, which, in the lungs, worsens respiratory failure.^2^^,^^10^^,^^31^^-^35

The endothelium has been studied previously in relation to other viral diseases, such as the human immunodeficiency virus (HIV). As with HIV, SARS-CoV-2 appears to have a direct endothelial aggression effect.^36^ Autopsy studies^33^^,^^34^ found evidence of direct infection by SARS-CoV-2 of the endothelial cell and diffuse inflammation.^35^ In addition to the impact of immunity, the endothelial dysfunction caused by SARS-CoV-2 would explain why patients with comorbidities of the blood vessels are at greater risk of developing severe COVID-19 and vice-versa.^4^^,^^28^

## FACTORS ASSOCIATED WITH SEVERE COVID-19

Elderly patients, males, and those with CVD and/or metabolic diseases are at greater risk of unfavorable progression of COVID-19. Cytokine “storms”, elevated acute phase proteins, and biomarkers of cardiac damage are characteristics of the severe form and predictors of death.^24^^,^^28^^,^^37^

The idea that inflammatory states are responsible for the onset or exacerbation of diseases is well-established. The association of inflammatory cells and their products with the pathophysiology of atherosclerosis is widely recognized and so is their relationship with the components of the metabolic syndrome (obesity, diabetes mellitus, and hypertension).^27^^,^^28^^,^^36^^,^^38^^,^^39^

Richardson et al.^40^ evaluated 5,700 electronic patient records from patients hospitalized with COVID-19 in six hospitals in New York and observed that 57% were hypertensive, 41% were obese, and 34% were diabetic. The most recent data in the global literature report greater mortality from COVID-19 in patients with these comorbidities. Also of note in this study from New York was that no deaths were reported among people under the age of 18 years. Although cardiometabolic diseases can have onset in childhood, they are considerably more prevalent in adults and the elderly.

The challenge is to understand why certain people can successfully recover their immunological homeostasis after suffering an insult and others do not.^2^^,^^10^^,^^12^^,^^14^^,^^19^^,^^20^ Possibly, some people are genetically susceptible to an imbalanced inflammatory response and, in these people, a single stimulus can have disastrous consequences, such as severe COVID-19, for example.^29^^,^^30^

SARS-CoV-2 activates the production of interleukin 12 (IL-12) and IFN-γ by dendritic cells, macrophages, and NK cells. These cytokines stimulate immature CD4+ T cells to differentiate into T_H_1 cells. The primary function of the T_H_1 phenotype is the production of IFN-γ and TNF-α. In addition to stimulating the T_H_1 response, IFN-γ plays a crucial role in the antiviral response as a macrophage activator and inducer of IgG production by B lymphocytes.^41^

Of the many chemokines and cytokines that exist, five (IL-1, IL-6, TNF-α, IL-8, and CCL2/MCP-1) are involved in diseases such as psoriasis, rheumatoid arthritis, and atherosclerosis. Some of the roles of these cytokines are well-established: increased cellular expression of adhesion molecules, chemotaxis of inflammatory cells, stimulation of cell differentiation and production of acute phase proteins, and proliferation of smooth muscle cells. All of these processes amplify the local and systemic inflammatory response, involving platelets and the fibrinolysis and coagulation systems.^2^^,^^14^^,^^22^^,^^38^^,^^42^

Just as in COVID-19, atherosclerosis involves a predominance of T_H_1 response, involving IFN-γ, TNF-α, and TNF-β, which amplify the inflammatory response. IFN-γ is considered one of the principal pro-atherogenic cytokines, promoting the participation of these cells in the inflammatory response by activating macrophages.^2^^,^^10^^,^^14^^,^^38^^,^^43^

Additionally, studies have demonstrated the production of IL-12 by monocytes in response to oxidized low density lipoprotein. These results prove that IL-12 expressed in atherosclerotic lesions can amplify responses of T_H_1 CD4+ T lymphocytes, considerably contributing to the immunopathology of atherosclerosis. Notwithstanding, this process can be highly regulated by the action of anti-inflammatory cytokines, such as IL-10.^2^^,^^5^^,^^10^^,^^14^^,^^22^^,^^38^^,^^44^ In view of the above, it is clear that amplification of the atherosclerotic process is triggered by a specific immunoresponse, which generates T_H_1 pathway cytokines, such as IL-12 and IFN-γ.^27^^,^^38^^,^^39^^,^^43^

In addition to the cytokines, positive acute phase proteins, particularly C-reactive protein, have also been targeted in several different studies of the pathogenesis of atherosclerosis. In addition to their classic role of opsonization of antigens, other interesting aspects have been investigated, such as their elevation in conditions of obesity, inflammation, metabolic syndrome, diabetes, and arterial hypertension, making them a predictive marker of risk of CVD.^10^^,^^22^^,^^44^

Cardiovascular diseases and metabolic syndrome are also consequences of immune system imbalances. People who have these diseases, even the youngest among them who have incipient atherosclerosis or metabolic syndrome, are more susceptible to the severe form of COVID-19, since they already have a hyperactive and dysregulated immune “territory”.

Considering that chronic inflammatory processes of the endothelium with a secondary autoimmune component and with specific immune responses against self-antigens are involved in the pathophysiology of atherosclerosis and the cardiometabolic diseases, we postulated that these individuals could have a more active expression of innate immune system cells ready to recognized SARS-CoV-2.^10^^,^^43^ After recognition, these cells would stimulate the production of IFN-γ by NK cells which, in turn, would induce macrophages to present viral antigens to T and B lymphocytes in an exaggerated and uncontrolled manner when compared to the cells of healthy individuals.

COVID-19 immunopathology is characterized by the elevation of IL-6 and TNF-α. These cytokines are products of activation of Toll 4 receptors (TLR4), which are components of innate immunity.^16^^,^^42^^,^^45^ A study conducted by computer simulation^16^ concluded that the SARS-CoV-2 spike protein (the same protein that binds to the ACE2 receptors) interacts with TLR4, thereby suggesting that this is the receptor responsible for recognition of SARS-CoV-2 by the immune system.

That TLR4 is involved in the pathogenesis of atherosclerosis is already well known. Several different types of cells present in atherosclerotic plaques express TLR4 and several proatherogenic ligands appear to activate TLR4. These receptors also participate in the pathophysiology of cardiometabolic diseases such as obesity and diabetes; TLR4 is involved in lipotoxicity and pancreatic beta cell dysfunction.^16^^,^^42^^,^^45^ Hyperexpression of TLR4 may even be genetically coded.^16^^,^^42^

Another mechanism probably responsible for worse progression in COVID-19 involves the ACE2 receptor. The reduction in ACE2 activity caused by SARS-CoV-2 has implications for CVD because they amplify dysregulation of the RAAS and immune system (Figure 1).^18^^,^^43^ Additionally, ACE2 receptors and dipeptidyl peptidase are enzymes that break down bradykinin, a vasoactive peptide. After SARS-CoV-2 binds to ACE2, the viral complex undergoes endocytosis, and the surface ACE2 is inhibited. As a result, the potential for breakdown of bradykinins is reduced.^18^^,^^43^ It has been speculated that excess bradykinin can complicate SARS-CoV-2 infection because of vasodilation effects, increased vascular permeability, and exacerbation of the coughing reflex.

ACE1 also breaks down bradykinin, and drugs that inhibit this enzyme (angiotensin-converting enzyme inhibitors [ACEi]) cause an increase in tissue bradykinin and can provoke coughing and angioedema in hypersensitive individuals. However, published studies have shown that the use of medications that block RAAS, such as ACEi and angiotensin receptor blockers (ARB), did not increase COVID-19 mortality and may even be a protective factor.^18^

The elderly population is at increased risk of death from COVID-19. Aging is a condition associated with inflammation, whereas the neonatal period is linked to an immature and anti-inflammatory immunoresponse.^25^ Men appear to be more susceptible than women to the severe form of COVID-19. Sexual differences (genetic, environmental, and hormonal) are reflected in differences in the immune system, resulting in variable responses to infection.^9^

A series of cases with immunodeficiencies also yielded interesting evidence. COVID-19 followed a mild clinical course in patients with agammaglobulinemia without B lymphocytes but progressed aggressively in common variable immunodeficiency. These results suggest mechanisms for possible therapeutic targets.^12^^,^^14^^,^^20^

## COAGULATION ABNORMALITIES IN COVID-19

Hyperinflammatory states cause platelet activation, endothelial dysfunction, and blood stasis, conditions that are directly related to venous and arterial thrombosis.^46^^-^48 Coagulopathy in severe COVID-19 infection is similar to sepsis-induced coagulopathy (SIC), characterized by disseminated intravascular coagulation (DIVC) and thrombotic microangiopathy. Furthermore, since the lungs are the organ most affected in COVID-19, hypoxemia is a risk factor for thrombosis.^31^

Adequate pulmonary function is totally dependent on an intact alveolar-capillary membrane. The SARS-CoV-2 virus provokes SARS. This syndrome is the body’s response to severe pulmonary aggression that triggers a series of physiological defense mechanisms, culminating in a form of autoaggression. This amplifies the inflammatory response, which can progress to Systemic Inflammatory Response Syndrome and multiple organ failure.^5^^,^^10^^,^^21^^,^^34^^,^^44^

Authors have also observed a build-up of insoluble fibrin in the alveolar space in SARS, due to incomplete fibrinolysis. One hypothesis is that the fibrinogen leaks from plasma because of diffuse alveolar damage and is not completely eliminated due to hypofibrinolysis. This insoluble fibrin will then contribute to pulmonary fibrosis and its negative outcomes.^49^^-^51

It should be understood that alveolar thrombotic microangiopathy is a primary thrombosis triggered by COVID-19 and differs from pulmonary arterial thrombosis secondary to classic venous thromboembolism (VTE). In classic pulmonary embolism (PE), the thrombus is actually an embolus that migrates, primarily from the deep veins of the lower limbs, whereas in COVID-19 the primary thromboses apparently predominantly originate in pulmonary capillaries.^34^ To date, there are no conclusive studies of the incidence of VTE in COVID-19.

Thrombotic complications appear to emerge as an important issue in patients infected by COVID-19. A retrospective French study suggested that systematic investigation of VTE and early therapeutic anticoagulation are warranted in patients with severe COVID-19 in the intensive care unit (ICU). Eight of a total of 26 patients were given prophylactic anticoagulation and 18 were given full anticoagulation, depending on their level of VTE risk. Of these patients, 69% exhibited VTE; 100% in the group given prophylactic anticoagulation and 56% in the group given full anticoagulation (p = 0.03). Surprisingly, we realized that the incidence of VTE was still high even in those given full anticoagulation. Moreover, there were six cases of PE among these patients.^49^

According to the experience accumulated over the 6 months since the disease emerged, the most important coagulation abnormalities observed were: increased generation of thrombin, D-dimers, and fibrinogen (initially), and prolongation of the prothrombin time (PT); with reduced fibrinolysis and platelet counts.^3^^,^^8^^,^^31^^,^^48^^,^^49^ These abnormal coagulation parameters have been observed in COVID-19 since the initial reports from China. Among the first 99 patients hospitalized in Wuhan, it was observed that 6% exhibited prolonged activated partial thromboplastin time (TTPa), 5% had elevated PT, and 36% had high D-dimers, in addition to increases in biomarkers of inflammation, including IL-6, erythrocyte sedimentation rate (ESR), and C-reactive protein. Thrombocytopenia occurred in 12% of cases, five patients had other coinfections (one bacterial and four fungal) and four patients had septic shock.^9^^,^^52^^,^^53^

D-dimer levels can help with early recognition of patients at greater risk of death, warning of the need for increased care. Preliminary data show that, in patients with severe COVID-19, the anticoagulant treatment appears to be associated with lower mortality in the subset that fulfills the criteria for SIC or has extremely elevated D-dimer levels.^31^^,^^51^

Thrombocytopenia is relatively common among patients with COVID-19 and is associated with an increased risk of hospital mortality. The lower the platelet count, the higher the mortality. However, many patients with severe COVID-19 do not yet exhibit this finding when admitted to the ICU.^25^^,^^46^^,^^47^^,^^51^

Prolongation of the PT is also an important marker of severity. In situations with thrombocytopenia and a prolonged PT, it may be useful to assay fibrinogen, as recommended by the International Society of Thrombosis and Haemostasis (SITH), to evaluate the possibility of DIVC.^51^

The SITH has produced diagnostic criteria for DIVC and has developed and validated criteria for SIC. The coagulation abnormalities associated with SIC are less severe and occur later than those associated with DIVC.^51^

Tang et al. found that 71% of patients who died from COVID-19 fulfilled the SITH criteria for DIVC, whereas only 0.6% of the survivors had DIVC. These researchers also observed a statistically significant increase in D-dimer levels and PT and a reduction in fibrinogen levels from days 10 to 14 of the disease among those who did not survive.^52^

A retrospective study from China suggests that the endothelial dysfunction induced by COVID-19 is responsible for the excessive thrombin production and fibrinolysis deficiency, which indicates a hypercoagulable state.^9^^,^^15^ A total of 449 individuals were studied, the majority were men (59.7%), with a mean age of 65.1±12.0 years and one or more chronic diseases (60.6%). Of these, 99 (22.0%) were given anticoagulant for at least 7 days, 94 of whom were given low molecular weight heparin (LMWH), enoxaparin 40-60 mg/day, and five were given 10,000-15,000 UI/day of unfractionated heparin (UFH).^9^

The study assessed factors predictive of mortality, comparing survivors and non-survivors of COVID-19, with or without treatment with heparin. No significant difference in mortality at 28 days was observed between those given and not given heparin (30.3% vs. 29.7%, p = 0.910). However, anticoagulation with heparin was associated with lower mortality among patients with SIC score ≥ 4 (40.0% vs. 64.2%, p = 0.029), but not among those with SIC score < 4 (29.0% vs. 22.6%, p = 0.419). In patients with D-dimers elevated to more than six times the upper limit of normality, the use of heparin also reduced mortality by around 20% (32.8% *vs*. 52.4%, p = 0.017). Hemorrhagic complications were rare and generally mild.^9^

This study prompted the creation of a flow diagram to guide anticoagulation decisions in COVID-19 according to disease severity criteria, SIC score, and/or D-dimer levels. The increased risk of thrombi can also be seen in the arteries, not just the veins. Depending on the vessel affected, there may be different clinical manifestations: stroke, mesenteric ischemia, acute myocardial infarction, or lower limb arterial occlusion.^9^^,^^51^^,^^52^

Oxley et al. reported hospital admission of five patients with SARS-CoV-2 and severe stroke over a period of 2 weeks. According to the publication, this observation constitutes a significant increase in patients under the age of 50 years suffering severe strokes compared to admissions data for the preceding 12 months. Two of these patients had no risk factors or previous history of stroke, one had dyslipidemia and hypertension, one was diagnosed with diabetes at the service, and the last had a history of stroke and diabetes.^54^

Compatible with the hypothesis of vascular aggression, some cases with characteristics of shock toxic syndrome or Kawasaki disease were reported by the United Kingdom Paediatric Intensive Care Society, the Spanish Pediatrics Association and the Italian Society of Pediatricians.^55^ Kawasaki disease is a vasculitis of the medium vessels and can be triggered by COVID-19. Its etiology is unknown as yet, but epidemiological and clinical presentation suggest that the cause is an abnormal infection or immunoresponse to a pathogen in genetically predisposed children.^55^

## TREATMENT

In line with the pathophysiology described in the previous sections, and respecting the proposed scope of the article, we will cover the treatments formulated in attempts to improve the resolution of severe forms of the disease. To date, there is no specific treatment with proven efficacy against COVID-19. Therapeutic strategies are based on early recognition of complications and optimized support to relieve symptoms.

A search made on June 25, 2020, on clinicaltrials.gov using the keywords “Covid and treatment” returned 1,563 registered studies, 366 in phases III and IV. Several antiviral and immunomodulatory treatments are being tested in the different stages of COVID-19 and results will be published over the coming months. Until a vaccine is available, we need to increase understanding of what leads some patients to suffer such poor outcomes, to avoid fatal outcomes using the measures and treatments available.

**Figure 2:**
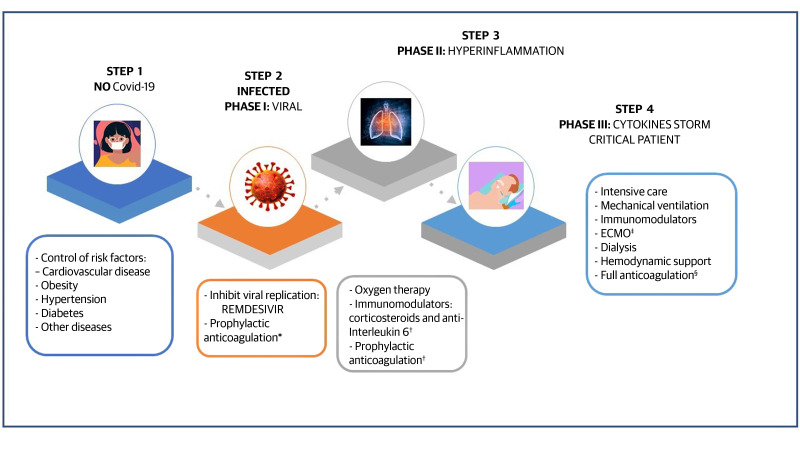
Strategies for treatment of COVID-19 according to clinical disease phase. *Studies in progress; ^†^Dose adjustment by weight and creatinine clearance (ClCr); ^‡^ECMO (extracorporeal membrane circulation); ^§^When there is a strong clinical suspicion of venous thromboembolism or confirmation of pulmonary embolism or proximal deep venous thrombosis (adjustment to weight and CrCl).

In response to the urgent need to identify effective treatments for COVID-19 in controlled and randomized studies, certain agents are being used in various countries on the basis of evidence obtained in vitro. The results from other viral diseases are being extrapolated and treatments are also being chosen based on observational studies and small clinical trials. Figure 2 illustrates treatment strategies by the disease phase.

The risk factors related to unfavorable disease outcomes can be divided into modifiable and nonmodifiable. Male sex and advanced age are nonmodifiable factors. However, the most elderly people should be isolated as a priority, particularly if they have chronic inflammatory diseases.^25^ Active smokers also require greater care, since the lungs are the organ in which the disease’s complications set in from phase II onwards and because smoking is an important risk factor for atherosclerosis.^1^

Patients with CVD and metabolic syndrome and its principal components (obesity, diabetes, and hypertension) merit great care.^24^ Therapeutic management of these factors appears to be a fundamental measure for reducing the reactivity of the endothelium and, as a result, reducing its vulnerability to COVID-19.^40^ Optimization of drug treatment with the use of hypoglycemics, antihypertensives, antihyperlipidemics (primarily statins) and platelet antiaggregants such as acetylsalicylic acid (AAS) can help stabilize the endothelium and reduce its reactivity.

Drugs such as ACEi and ARB help to balance the RAAS. As discussed above, this system appears more dysregulated in COVID-19. Additionally, observational studies in patients hospitalized with COVID-19 have suggested that the risk of death is lower among patients on these medications, especially those on ACEi.^18^

Once infected with SARS-CoV-2, the first step is the early use of drugs that can prevent replication and facilitate viral clearance. We do not yet have an ideal drug, but studies are ongoing all over the world with antimalarials, antivirals, antibacterials, and antiparasitics. There are unpublished reports of success with a “hit early and hit hard” approach. This hypothesis is being tested in multicenter clinical trials in Brazil and abroad with some of these drug classes.^56^^,^^57^

In the midst of the pandemic, conduct is being adjusted as knowledge evolves and much of what is already routine treatment for critical patients is being applied to the treatment of COVID-19. The most important complications of COVID-19 are SARS, SIC, VTE, and DIVC. All of these conditions are well-known in sepsis and in COVID-19 they appear to be the consequence of the dysregulated inflammatory response.

SARS is the first major complication and specific treatment protocols for it exist.^4^^,^^7^ Measures such as supplemental oxygen, support in the ICU, and mechanical ventilation are fundamentally important.

While still in the hyperinflammatory phase, drugs that inhibit or reduce the effects of proinflammatory cytokines appear very relevant. IL-6 inhibitors and also glucocorticoids can avert or minimize the cytokine storm. New medications that modulate inflammatory response are fundamental in this phase to avoid excessive inflammation, which attacks the endothelium and many different organs with great intensity and has the potential to cause multiple organ failure and death.^58^

Studies have shown an increased risk of deep venous thrombosis (DVT) and PE in patients hospitalized with COVID-19^49^ and it is important to monitor these patients using clinical scores for DVT and PE.^59^ In relation to VTE, studies indicate that hospitalized patients should be given pharmacological thromboprophylaxis with LMWH or fondaparinux (preferably with UFH), unless the risk of bleeding outweighs the risk of thrombosis.^31^^,^^47^^,^^51^

Adjusting the heparin dose for obesity and for patients with renal failure is recommended (Table 1).^25^^,^^51^ We do not yet have studies that can support the use of anticoagulants in patients without a clinical need for hospital admissions for COVID-19 or the use of intermediate anticoagulation dosages, without at least a strong suspicion of VTE. Studies are ongoing into the use of anticoagulants in the different phases of COVID-19. There is a tendency to suggest that prophylaxis should be maintained after hospital discharge, because of the hypercoagulable state triggered by the disease.^50^^,^^60^^-^62

**Table 1:** Adapted summary of consensus recommendations for antithrombotic treatment during the COVID-19 pandemic.

**COVID-19 phases**	**Antithrombotic treatments**
OUTPATIENTS	· Encourage walking;
**PHASE I**	· Assess risk of VTE vs. hemorrhagic risk;
	· Consider pharmacological prophylaxis if there is a high thrombotic risk without high hemorrhagic risk;
	· Patients on antithrombotic treatment: maintain treatment;
	· Patients on antivitamin K without adequate control: suggest transition to direct oral anticoagulant or enoxaparin.
HOSPITALIZED	· Assess risk of VTE vs. hemorrhagic risk;
**PHASE II and III WITHOUT DIVC**	· Initiate prophylactic dosage LMWH - subcutaneous enoxaparin 40 mg once a day* (if contraindicated, use mechanical prophylaxis).
HOSPITALIZED	· Pharmacological prophylaxis if no risk of bleeding;
**PHASE III**	· No recommendation for intermediate or full dosage of LMWH or routine UFH;
**WITH DIVC**	· Users of full anticoagulation: maintain treatment. Consider reduction of dose according to risk of bleeding;
	· Users of double platelet antiaggregation: assess individualized risk/benefit of suspension or maintenance. If platelets > 50,000, maintain double treatment; platelets from 25,000 to 50,000, maintain one antiplatelet drug; platelets < 25,000, withdraw antiaggregation;
	· After hospital discharge: assess risk of VTE and consider pharmacological prophylaxis for up to 45 days. Encourage physical activity and walking.

* Avoid enoxaparin if creatinine clearance < 30 mL/min; choose UFH 5,000 UI, 2 or 3 times/day. DIVC = disseminated intravascular coagulation; LMWH = low molecular weight heparin; UFH = unfractionated heparin; VTE = venous thromboembolism.

There is a risk of bleeding in anticoagulant patients, particularly so as the disease worsens. The profile of DIVC in COVID-19 is initially thrombotic, but it can progress to hemorrhagic as the disease progresses. It is very important to monitor platelet counts and serum fibrinogen levels and also to calculate the risk of bleeding scores such as IMPROVE (International Medical Prevention Registry on Venous Thromboembolism). Patients with an IMPROVE score < 7 are at a low risk of bleeding and should be kept on pharmacological prophylaxis. Cases with scores > 7 are at high risk of bleeding and mechanical prophylaxis is indicated.^63^

With relation to the treatment of COVID-19, the therapeutic strategy is based on early recognition of complications and optimized support for the relief of symptoms. To date, there is no specific treatment with proven efficacy against COVID-19. Figure 2 illustrates the principal therapeutic recommendations by the clinical phase of COVID-19.

## CONCLUSIONS

COVID-19 immunopathology appears to share the same TLR4 receptor as CVD and metabolic syndrome. It is possible that inappropriate activation of this receptor is the factor responsible for the exaggerated immunoresponse seen in patients with the severe form of SARS-CoV-2.

In summary, while we await a vaccine, the best treatment for COVID-19 is perhaps that which encompasses treatments that improve patients’ cardiovascular and metabolic conditions, in addition to medications that reduce viral replication, hyperinflammation, and the risk of thrombosis.
