# Supplementary material for: Severe COVID-19: understanding the role of immunity, endothelium, and coagulation in clinical practice
Source: J Vasc Bras. 2020 Nov 16;19:e20200131. doi: 10.1590/1677-5449.200131 (PMC8218014; doi:10.1590/1677-5449.200131)
Supplement: Translation

# Translation: COVID-19 grave: entenda o papel da imunidade, do endotélio e da coagulação na prática clínica

**Authors:** Simone Cristina Soares Brandão, Emmanuelle Tenório Albuquerque Madruga Godoi, Júlia de Oliveira Xavier Ramos, Leila Maria Magalhães Pessoa de Melo, Emanuel Sávio Cavalcanti Sarinho

**Affiliations:** 1 Universidade Federal de Pernambuco – UFPE, Recife, PE, Brasil.; 2 Serviço de Hematologia de São José dos Campos, São José dos Campos, SP, Brasil.

**Keywords:** COVID-19, endotélio, imunidade, aterosclerose, trombose

## Abstract

O SARS-CoV-2 é o responsável pela pandemia da COVID-19. O sistema imunológico é fator determinante no combate à infecção viral e, quando atua equilibrada e eficientemente, a doença é autolimitada e benigna. Uma parcela significativa da população, porém, apresenta resposta imune exacerbada. Os indivíduos diabéticos, hipertensos, obesos e com doenças cardiovasculares, infectados pelo vírus, apresentam maior chance de progredir para formas graves. Essas doenças estão relacionadas a processos inflamatórios crônicos e disfunção endotelial. Os receptores do tipo Toll estão presentes nas células de defesa e participam da imunopatologia de doenças cardiovasculares e metabólicas, levando à produção de citocinas pró-inflamatórias quando ativados. Devido à ação viral e à hiperativação do sistema imune, estados de hiperinflamação, hiperativação plaquetária, disfunção endotelial e hipercoagulabilidade são desenvolvidos, predispondo a tromboses venosas e arteriais. Discutiremos sobre a interação entre a COVID-19, a imunidade, o endotélio e a coagulação, como também sobre as possíveis causas de doenças cardiometabólicas impactarem negativamente na evolução da COVID-19.

## INTRODUÇÃO

O coronavírus 2 da síndrome respiratória aguda grave (SARS-CoV-2) é responsável pela doença COVID-19 (*COronaVIrus Disease* 2019). Os coronavírus causam surtos que ameaçam a saúde, incluindo o da síndrome respiratória aguda grave (SARS), que ocorreu na China em 2002, a síndrome respiratória do Oriente Médio (MERS), em 2012, e a atual pandemia de COVID-19^1^^-^3.

O SARS-CoV-2 foi identificado em pacientes com pneumonia atípica, caracterizada por febre, tosse seca e dispneia progressiva^1^^,^^4^^-^6. Os dados iniciais, provenientes da China, mostram que 81% dos casos de COVID-19 apresentam sintomas leves a moderados, incluindo pacientes sem pneumonia ou com pneumonia leve. Entre os pacientes, 14% evoluem para uma doença grave e 5% tornam-se doentes críticos com falência respiratória e de múltiplos órgãos, com altíssimo risco de morte^1^^,^^7^^-^9.

A grande questão é compreender por que alguns indivíduos progridem para a forma grave da COVID-19. Estudos têm revelado uma relação significativa entre gravidade da doença e marcadores imunes. Foi sugerido que, durante a resposta ao SARS-CoV-2, a desregulação imunológica e o alto nível de citocinas pró-inflamatórias poderiam ser a causa principal de lesão tecidual. Além do mais, indivíduos com doenças cardiovasculares (DCV) e metabólicas apresentam maior risco de morte pela COVID-19^2^^,^^10^^-^12.

Assim, diante desta pandemia, vários desafios foram impostos à medicina, uma vez que a urgência em salvar vidas é uma necessidade. Sabemos que a resposta imune interage intimamente com o endotélio e o sistema de coagulação e que, na fisiopatologia das DCV e metabólicas, processos inflamatórios crônicos estão envolvidos. Nesta revisão, discutiremos sobre a interação entre a COVID-19, a imunidade, o endotélio e a coagulação, como também sobre os possíveis aspectos que levam as doenças cardiometabólicas a impactarem negativamente em casos de COVID-19. O objetivo é provocar uma maior reflexão sobre a influência desses fatores na abordagem terapêutica dessa nova doença.

## COVID-19, RESPOSTA IMUNE E FASES DA DOENÇA

O sistema imunológico atua na defesa contra agentes infecciosos. Essa função é mediada por reações iniciais da imunidade inata e tardias da imunidade adaptativa, que são determinantes no combate às infecções virais. Na COVID-19, uma resposta inflamatória eficiente e equilibrada permite uma evolução autolimitada e benigna da doença. A forma grave ocorre numa parcela de pacientes que apresenta uma resposta imune exacerbada ao SARS-CoV-2^1^^,^^13^^-^15.

O SARS-CoV-2, um membro da família de betacoronavírus, possui RNA de fita simples com proteínas estruturais típicas, envolvendo as proteínas de envelope (proteína E), membrana (proteína M), nucleocapsídeo (proteína N) e espícula (proteína S, do inglês *spike*), responsáveis pela infectividade viral^16^^,^^17^. As proteínas S na superfície do SARS-CoV-2 se ligam aos receptores humanos ECA2 (enzima conversora de angiotensina 2), uma proteína transmembrana, os quais, por sua vez, transferem seu material genético para dentro da célula e, em seguida, iniciam seu processo de replicação^16^^-^18.

As células humanas, quando infectadas por vírus, são reconhecidas pelos sistemas imunes tanto inato como adaptativo, que iniciam a produção de citocinas. Dentre as principais citocinas produzidas, destacam-se o fator de necrose tumoral alfa (TNF-α) e o interferon-gama (IFN-γ). O primeiro é responsável pela ativação neutrofílica, promoção da coagulação e atuação a nível central para produção de febre; o segundo induz atividade macrofágica de destruição do patógeno e amplia a liberação de citocinas (pró-inflamatórias, pró-fibróticas e regulatórias da resposta imune)^2^^,^^10^^,^^14^^,^^19^^-^21.

Adicionalmente, a ECA2 é uma carboxipeptidase capaz de converter a angiotensina 2 em angiotensinas 1-7. Essa enzima é homóloga à ECA1, mas desempenha um papel de contrapeso no sistema renina-angiotensina-aldosterona (SRAA)^16^^,^^18^^,^^22^. Quando o vírus se liga ao receptor ECA2, reduz a produção de angiotensinas 1-7, podendo desencadear uma série de complicações cardiovasculares e pró-inflamatórias (Figura 1)^18^.

**Figura 1:** Ações da angiotensina 1-7, subproduto da angiotensina I, e consequências do seu bloqueio. A enzima conversora de angiotensina 1 (ECA1) e a enzima conversora de angiotensina 2 (ECA2) agem na angiotensina I e II. A proteína S do SARS-CoV-2 (coronavírus 2 da síndrome respiratória aguda grave) se liga ao receptor ECA2 da célula humana, reduzindo sua atividade enzimática. AT1R = receptor da angiotensina II tipo 1; AT2R1 = receptor da Angiotensina II tipo 2; MASR = receptor MAS da angiotensina 1-7.

As fases da COVID-19 parecem estar relacionadas à intensidade da resposta imune. Quando existe resposta inflamatória adequada, os pacientes não progridem da fase I e ocorre resolução da infecção. À medida que existe uma resposta imune exacerbada, a doença vai evoluindo para as fases II e III.

A forma leve (fase I) geralmente se caracteriza pela presença de febre, tosse seca e fadiga. Outras manifestações são diarreia, mialgia, cefaleia, odinofagia, anosmia, ageusia e coriza. Já a forma grave (fase II) se caracteriza por dispneia, taquipneia, queda na saturação de oxigênio e infiltrado pulmonar ao raio X ou tomografia computadorizada de tórax. Os casos críticos (fase III) apresentam sinais de choque circulatório, falência respiratória e disfunção de múltiplos órgãos^2^^,^^4^^,^^6^^,^^7^^,^^23^^-^25.

Uma resposta imune modulada contra o SARS-CoV-2 parece ser fundamental para a resolução da COVID-19. Ela se dá pela coordenada produção de citocinas pró-inflamatórias (IFN-γ e TNF-α) e anti-inflamatórias [interleucina-10 (IL-10)], a qual, junto com a atuação celular e das imunoglobulinas (Ig), age a fim de atingir o máximo potencial de combate ao vírus^14^^,^^19^^,^^20^.

No manejo do paciente, é de grande relevância definir-se a fase da doença através de anamnese adequada^6^^,^^7^. Uma síndrome de inflamação pulmonar e extrapulmonar, chamada de linfohistiocitose hemofagocítica secundária (LHHs), pode estar presente na fase III. A LHHs se caracteriza por hiperativação imunológica devido à eliminação inadequada de macrófagos ativados pelas células *natural killer* (NK) e pelos linfócitos T citotóxicos, resultando em produção excessiva de citocinas pró-inflamatórias^2^. Em sequência, há uma “tempestade” de citocinas e uma exacerbação dos mecanismos inflamatórios. Esse quadro pode causar o óbito^2^^,^^25^.

Desta forma, os pacientes com quadros graves da COVID-19 devem ser rastreados para a hiperinflamação, utilizando marcadores laboratoriais e o HScore^26^. Esse escore foi a primeira pontuação validada para o diagnóstico de síndrome hemofagocítica reativa. A pontuação foi baseada no maior conjunto de dados de pacientes adultos com suspeita de síndrome hemofagocítica reativa e é composta por nove variáveis clínicas, biológicas e citológicas, adequadamente pesadas. As pontuações maiores que 169 do HScore são 93% sensíveis e 86% específicas para LHHs^26^.

A intensidade da resposta imune ao SARS-CoV-2 interfere negativamente com a função endotelial e as doenças preexistentes ligadas ao endotélio são fatores associados à gravidade da COVID-19. Essa rede de comunicação deixa evidente por que, na forma grave, o sistema imune/hiperinflamação, o endotélio e a coagulação estão ciclicamente envolvidos.

## RESPOSTA DO ENDOTÉLIO À COVID-19

O endotélio é um órgão ativo, com funções endócrina, parácrina e autócrina, indispensáveis para a regulação do tônus e a manutenção da homeostase vascular^5^^,^^27^. Na COVID-19, o recrutamento de células imunes, pela agressão direta viral ao endotélio ou imunomediada, pode resultar em disfunção endotelial generalizada associada a apoptose^5^^,^^8^^,^^28^^-^30.

Nos casos de maior gravidade, o óbito acontece por falência de múltiplos órgãos. Estudos histológicos *post mortem* revelaram um quadro de endotelite linfocítica nos pulmões, coração, rins e fígado, bem como necrose celular e presença de microtrombos, que, nos pulmões, piora a insuficiência respiratória^2^^,^^10^^,^^31^^-^35.

O endotélio já foi estudado em outras doenças virais, como no vírus da imunodeficiência humana (HIV). Assim como o HIV, o SARS-CoV-2 parece ter um efeito direto de agressão endotelial^36^. Em estudos de autópsias^33^^,^^34^, foram encontradas evidências de infecção direta do SARS-CoV-2 na célula endotelial e inflamação difusa^35^. Adicionalmente ao impacto da imunidade, a disfunção endotelial causada pelo SARS-CoV-2 justificaria por que pacientes com comorbidades relacionadas aos vasos sanguíneos têm maior risco de desenvolver a COVID-19 grave e vice-versa^4^^,^^28^.

## FATORES ASSOCIADOS À COVID-19 GRAVE

Pacientes idosos, do sexo masculino ou com DCV e/ou metabólicas têm apresentado maior chance de evolução desfavorável da COVID-19. “Tempestades” de citocinas, proteínas de fase aguda elevadas e biomarcadores de lesão cardíaca são características da forma grave e preditivos de morte^24^^,^^28^^,^^37^.

A ideia de que estados inflamatórios sejam responsáveis pela instalação de doenças ou pelo seu agravamento encontra-se bem estabelecida. A associação de células inflamatórias e seus produtos é bem reconhecida na fisiopatologia da aterosclerose, como também nos componentes da síndrome metabólica (obesidade, diabetes *mellitus* e hipertensão)^27^^,^^28^^,^^36^^,^^38^^,^^39^.

Richardson et al.^40^ avaliaram 5.700 prontuários eletrônicos de pacientes hospitalizados com COVID-19 em seis hospitais de Nova York e observaram que 57% eram hipertensos, 41% eram obesos e 34% eram diabéticos. Os dados mais recentes da literatura mundial relatam a maior mortalidade pela COVID-19 em pacientes com essas comorbidades. Ademais, destaca-se que, nesse estudo novaiorquino, não foram relatadas mortes em menores de 18 anos. Embora as doenças cardiometabólicas possam se iniciar na infância, elas são expressivamente mais prevalentes nas fases adulta e senil.

O desafio é entender por que certas pessoas conseguem restaurar a homeostase imunológica com sucesso após sofrerem um insulto e outras não^2^^,^^10^^,^^12^^,^^14^^,^^19^^,^^20^. Possivelmente, algumas pessoas são geneticamente suscetíveis a uma resposta inflamatória desequilibrada e, nessas pessoas, um único estímulo pode levar a consequências desastrosas, a exemplo da COVID-19 grave^29^^,^^30^.

O SARS-CoV-2 ativa a produção de interleucina 12 (IL-12) e IFN-γ por células dendríticas, macrófagos e NK. Essas citocinas estimulam as células T CD4+ imaturas a diferenciarem em células T_H_1. A principal função do fenótipo T_H_1 é a produção de IFN-γ e TNF-α. O IFN-γ, além de atuar estimulando a resposta T_H_1, possui papel crucial na resposta antiviral como ativador de macrófagos e produtor de IgG pelos linfócitos B^41^.

Dentre as várias quimiocinas e citocinas existentes, cinco (IL-1, IL-6, TNF-α, IL-8 e CCL2/MCP-1) estão envolvidas em doenças como psoríase, artrite reumatoide e aterosclerose. Alguns papéis já bem estabelecidos por essas citocinas são: aumento da expressão celular de moléculas de adesão, quimiotaxia de células inflamatórias, estímulo à diferenciação celular e à produção de proteínas de fase aguda e proliferação de células musculares lisas. Todos esses processos amplificam a resposta inflamatória local e sistêmica, envolvendo as plaquetas e os sistemas de fibrinólise e de coagulação^2^^,^^14^^,^^22^^,^^38^^,^^42^.

Na aterosclerose, assim como na COVID-19, existe predominância de resposta T_H_1, envolvendo IFN-γ, TNF-α e TNF-β, que amplificam a resposta inflamatória. A IFN-γ é considerada uma das principais citocinas pró-aterogênicas que, por ativar macrófagos, favorece a participação dessas células na resposta inflamatória^2^^,^^10^^,^^14^^,^^38^^,^^43^.

Adicionalmente, estudos mostram a produção de IL-12 por monócitos em resposta a lipoproteína de baixa densidade oxidada. Esses resultados provam que a IL-12, por sua expressão nas lesões ateroscleróticas, pode levar à amplificação de respostas de linfócitos T CD4+ do tipo T_H_1, contribuindo consideravelmente na imunopatologia da aterosclerose. Todavia, esse processo pode apresentar-se altamente regulado pela ação das citocinas anti-inflamatórias, como a IL-10^2^^,^^5^^,^^10^^,^^14^^,^^22^^,^^38^^,^^44^. Sendo assim, diante do exposto, torna-se evidente que uma amplificação do processo aterosclerótico ocorre a partir de uma resposta imune específica, que gera citocinas da via T_H_1, como a IL-12 e o IFN-γ^27^^,^^38^^,^^39^^,^^43^.

Além das citocinas, as proteínas positivas de fase aguda, principalmente a proteína C reativa, são alvos de vários estudos na patogênese da aterosclerose. Além de exercer o papel clássico de opsonização de antígenos, outros aspectos interessantes são investigados, como o seu aumento nas condições de obesidade, inflamação, síndrome metabólica, diabetes e hipertensão arterial, colocando-a como um marcador de risco preditivo para DCV^10^^,^^22^^,^^44^.

As DCV e a síndrome metabólica são também consequências de desequilíbrio no sistema imunológico. As pessoas com essas doenças, até mesmo as mais jovens com aterosclerose incipiente ou síndrome metabólica, estariam mais suscetíveis à forma grave da COVID-19, uma vez que já possuem um “terreno” imune hiperativo e desregulado.

Considerando que, na aterosclerose e nas doenças cardiometabólicas, os processos inflamatórios crônicos do endotélio, com um componente autoimune secundário e com respostas imunes específicas de antígeno próprio, estão envolvidos na sua fisiopatologia, postulamos que esses indivíduos poderiam expressar mais ativamente células do sistema imune inato prontas para reconhecer o SARS-CoV-2^10^^,^^43^. Após o reconhecimento, essas células estimulariam a produção de IFN-γ pelas células NK que, por sua vez, induziriam a apresentação pelos macrófagos de antígenos virais aos linfócitos T e B, de forma exacerbada e descontrolada, quando comparadas às células de indivíduos saudáveis.

A imunopatologia da COVID-19 se caracteriza por elevação de IL-6 e TNF-α. Essas citocinas são produtos de ativação do receptor do tipo Toll 4 (TLR4), que faz parte da imunidade inata^16^^,^^42^^,^^45^. Em um estudo por simulações computacionais^16^, concluiu-se que a proteína *spike* do SARS-CoV-2, a mesma proteína que se liga aos receptores ECA2, interage com o TLR4, sugerindo, assim, que esse receptor seria o responsável pelo reconhecimento do sistema imune ao SARS-CoV-2.

O envolvimento do TLR4 na patogênese da aterosclerose é bem conhecido. Diferentes tipos de células presentes na placa aterosclerótica expressam TLR4 e vários ligantes pró-aterogênicos parecem ativar o TLR4. As doenças cardiometabólicas, como obesidade e diabetes, também exprimem na sua fisiopatologia a participação desses receptores, estando os TLR4 envolvidos na lipotoxicidade e disfunção de células beta pancreáticas^16^^,^^42^^,^^45^. A hiperexpressão do TLR4 pode ser inclusive geneticamente codificada^16^^,^^42^.

Outro provável mecanismo responsável pela pior evolução da COVID-19 envolve o receptor ECA2. A redução da atividade da ECA2 pelo SARS-CoV-2 tem implicações nas DCV por potencializarem a desregulação do SRAA e do sistema imune (Figura 1)^18^^,^^43^. Adicionalmente, os receptores de ECA2 e dipeptidil peptidase são enzimas que decompõem a bradicinina, um peptídeo vasoativo. Após a ligação do SARS-CoV-2 ao ECA2, o complexo viral sofre endocitose, e o ECA2 de superfície é inibido. Por conseguinte, ocorre redução do potencial de decomposição das bradicininas^18^^,^^43^. Foi especulado se o excesso de bradicinina poderia complicar a infecção pelo SARS-CoV-2 devido aos efeitos da vasodilatação, do aumento da permeabilidade vascular e da exacerbação do reflexo da tosse.

A ECA1 também degrada a bradicinina, e as drogas que inibem essa enzima [inibidores da enzima conversora da angiotensina (IECA)] levam a um aumento na bradicinina tecidual e podem provocar tosse e angioedema em indivíduos hipersensíveis. Entretanto, estudos já publicados mostraram que o uso de medicações que bloqueiam o SRAA, como os IECA e os bloqueadores do receptor da angiotensina (BRA), não causou aumento de mortalidade pela COVID-19, podendo inclusive ser um fator de proteção^18^.

A população idosa apresenta maior risco de morte pela COVID-19. O envelhecimento é uma condição associada à inflamação, enquanto o período neonatal está relacionado a uma resposta imune imatura e anti-inflamatória^25^. Os homens parecem ser mais suscetíveis à forma grave da COVID-19 em relação às mulheres. Diferenças sexuais (genéticas, ambientais e hormonais) refletem em diferenças no sistema imune, levando a respostas variáveis à infecção^9^.

Outra evidência interessante foi demonstrada por uma série de casos com imunodeficiências. A COVID-19 apresentou curso clínico leve em pacientes com agamaglobulinemia sem linfócitos B, enquanto se desenvolveu agressivamente na imunodeficiência comum variável. Esses resultados oferecem mecanismos para possíveis alvos terapêuticos^12^^,^^14^^,^^20^.

## ALTERAÇÕES NA COAGULAÇÃO NA COVID-19

Os estados hiperinflamatórios levam a ativação plaquetária, disfunção endotelial e estase sanguínea, condições diretamente relacionadas a trombose venosa e arterial^46^^-^48. A coagulopatia na infecção grave por COVID-19 é semelhante à coagulopatia induzida pela sepse (SIC, de *sepsis-induced coagulopathy*), caracterizada por coagulação intravascular disseminada (CIVD) e microangiopatia trombótica. Além disso, como os pulmões são prioritariamente afetados na COVID-19, a hipoxemia é um fator de risco para trombose^31^.

A função adequada do pulmão é totalmente dependente de uma membrana alvéolo-capilar íntegra. O vírus SARS-CoV-2 provoca a SARS. Essa síndrome é a resposta do organismo a uma agressão pulmonar grave que dispara uma série de mecanismos fisiológicos de defesa, culminando em uma forma de autoagressão. Assim, amplifica-se a resposta inflamatória que pode evoluir para a síndrome da resposta inflamatória sistêmica e disfunção de múltiplos órgãos^5^^,^^10^^,^^21^^,^^34^^,^^44^.

Autores também observam na SARS um acúmulo de fibrina insolúvel no espaço alveolar devido à fibrinólise incompleta. Uma hipótese é que o fibrinogênio extravasa do plasma pelo dano alveolar difuso e não é eliminado completamente pela hipofibrinólise. Assim, essa fibrina insolúvel vai contribuir para a fibrose pulmonar e seus desdobramentos negativos^49^^-^51.

Destaca-se que a microangiopatia trombótica alveolar é uma trombose primária desencadeada na COVID-19 e difere da trombose arterial pulmonar secundária ao tromboembolismo venoso (TEV) clássico. Na embolia pulmonar (EP) clássica, o trombo é na verdade um êmbolo que migra principalmente das veias profundas dos membros inferiores, enquanto na COVID-19, aparentemente, prevalece a trombose primária dos capilares pulmonares^34^. Até o momento, não há estudos conclusivos sobre a incidência de TEV na COVID-19.

As complicações trombóticas parecem emergir como uma questão importante em pacientes infectados pela COVID-19. Um estudo francês retrospectivo sugere a investigação sistemática para TEV e anticoagulação terapêutica precoce em pacientes de unidade de terapia intensiva (UTI) com COVID-19 grave. Do total de 26 pacientes, oito receberam anticoagulação profilática e 18 receberam plena, de acordo com risco de TEV. Desses pacientes, 69% apresentaram TEV, sendo em 100% no grupo da profilaxia e 56% no grupo com anticoagulação plena (p = 0,03). Surpreendentemente, percebemos que a incidência de TEV foi alta, mesmo nos plenamente anticoagulados. Além do mais, nessa casuística, foram relatados seis casos de EP^49^.

De acordo com a experiência acumulada nesses 6 meses da doença, as principais alterações observadas na coagulação foram: aumento na geração de trombina, dímero-D, fibrinogênio (inicialmente) e no tempo de protrombina (TP); e redução na fibrinólise e contagem de plaquetas^3^^,^^8^^,^^31^^,^^48^^,^^49^. Desde os relatórios iniciais da China, esses parâmetros anormais de coagulação foram observados na COVID-19. Entre os primeiros 99 pacientes hospitalizados em Wuhan, foi observado que 6% dos pacientes apresentaram elevação do tempo de tromboplastina parcial ativado (TTPa), 5%, do TP, 36%, do dímero-D, além de aumento em biomarcadores de inflamação, incluindo IL-6, velocidade de hemossedimentação (VHS) e proteína C reativa. Ocorreu trombocitopenia em 12% dos casos, cinco pacientes tiveram outras coinfecções (uma bacteriana e quatro fúngicas) e quatro pacientes tiveram choque séptico^9^^,^^52^^,^^53^.

O dímero-D pode ajudar no reconhecimento precoce de pacientes de maior risco de morte, alertando para mais cuidado. Dados preliminares mostram que, em pacientes com COVID-19 grave, a terapia anticoagulante parece estar associada a menor mortalidade na subpopulação que atende aos critérios de SIC ou com dímero-D acentuadamente elevado^31^^,^^51^.

A trombocitopenia é relativamente comum em pacientes com COVID-19 e está associada a risco aumentado de mortalidade hospitalar. Quanto menor a contagem de plaquetas, maior a mortalidade. Entretanto, muitos pacientes com COVID-19 grave ainda não apresentam esse achado à admissão em UTI^25^^,^^46^^,^^47^^,^^51^.

O alargamento do TP também é importante marcador de gravidade. No cenário de trombocitopenia e TP alargado, pode ser útil dosar o fibrinogênio, conforme recomenda a Sociedade Internacional de Trombose e Hemostasia (SITH) para avaliar a possibilidade de CIVD (DIC em inglês, *disseminated vascular coagulopathy*)^51^.

A SITH postulou critérios diagnósticos para a CIVD, bem como desenvolveu e validou critérios para SIC. As alterações da coagulação associadas à SIC, em relação às associadas à CIVD, são menos graves e ocorrem de forma mais precoce^51^.

Tang et al. identificaram que 71% dos pacientes que morreram de COVID-19 preenchiam os critérios da SITH para CIVD, enquanto, nos sobreviventes, apenas 0,6% tinham CIVD. Esses pesquisadores também observaram um aumento estatisticamente significante nos níveis de dímero-D e TP e uma diminuição nos níveis de fibrinogênio em não sobreviventes entre os dias 10 e 14 da doença^52^.

Um estudo retrospectivo chinês sugere que a disfunção endotelial induzida pela COVID-19 seja responsável pela geração de excesso de trombina e deficiência da fibrinólise, que indica um estado hipercoagulável^9^^,^^15^. Nesse escopo, foram estudados 449 indivíduos, sendo a maioria homens (59,7%), com idade média de 65,1±12,0 anos e uma ou mais doenças crônicas (60,6%). Desses, 99 (22,0%) receberam anticoagulante por pelo menos 7 dias, dos quais 94 receberam heparina de baixo peso molecular (HBPM), enoxaparina 40-60 mg/dia, e cinco receberam heparina não fracionada (HNF), 10.000-15.000 UI/dia^9^.

Nesse estudo, fatores preditivos de mortalidade foram avaliados comparando sobreviventes e não sobrevivente à COVID-19, com ou sem tratamento com heparina. Não foi encontrada diferença significante na mortalidade em 28 dias entre usuários e não usuários de heparina (30,3% *vs.* 29,7%, p = 0,910). Entretanto, a anticoagulação com heparina foi associada a menor mortalidade nos pacientes com escore SIC ≥ 4 (40,0% *vs.* 64,2%, p = 0,029), mas não naqueles com escore SIC < 4 (29,0% *vs.* 22,6%, p = 0,419). Nos pacientes com dímero-D elevado, acima de seis vezes o limite superior de normalidade, o uso de heparina reduziu também mortalidade em cerca de 20% (32,8% *vs*. 52,4%, p = 0,017). As complicações hemorrágicas foram raras e geralmente leves^9^.

Esse estudo motivou a criação de um fluxograma para decisão de terapia anticoagulante na COVID-19, segundo critérios de gravidade da doença, escore SIC e/ou valor de dímero-D. O aumento de risco de trombos também pode ser visto nas artérias e não apenas nas veias. De acordo com o vaso acometido, pode haver diferentes manifestações clínicas: acidente vascular encefálico (AVE), isquemia mesentérica, infarto agudo do miocárdio ou oclusão arterial de membros inferiores^9^^,^^51^^,^^52^.

Oxley et al. relataram a admissão hospitalar de cinco pacientes com SARS-CoV-2 e AVE grave, em um período de 2 semanas. De acordo com a publicação, esse número corresponde a um significativo aumento de pacientes com menos de 50 anos que apresentaram AVE grave quando comparado aos dados de admissão nos últimos 12 meses. Dentre os pacientes, dois não possuíam fatores de risco ou história pregressa de AVE, um possuía dislipidemia e hipertensão, um foi diagnosticado no serviço com diabetes e o último possuía história de AVE e diabetes^54^.

Corroborando com a hipótese de agressão vascular, alguns casos com características da síndrome do choque tóxico ou doença de Kawasaki foram relatados pela Associação de Pediatras de Cuidados Intensivos do Reino Unido, pela Associação Espanhola de Pediatria e pela Sociedade Italiana de Pediatras^55^. A doença de Kawasaki é uma vasculite de médios vasos e pode ter na COVID-19 seu gatilho. Sua etiologia ainda é desconhecida, mas a epidemiologia e a apresentação clínica sugerem como causa infecção ou resposta imunológica anormal a um patógeno em crianças geneticamente predispostas^55^.

## TRATAMENTO

Em consonância com a fisiopatologia que foi estudada nos tópicos anteriores e respeitando o escopo proposto para o presente artigo, abordaremos as propostas de tratamento na tentativa de melhor resolutividade das formas graves da doença. Até o momento, não existe tratamento específico que seja comprovadamente eficaz para a COVID-19. A estratégia terapêutica tem se baseado no reconhecimento precoce das complicações e no suporte otimizado para aliviar os sintomas.

Numa consulta realizada em 25 de junho de 2020 no clinicaltrials.gov com os descritores “*Covid and treatment*”, encontramos 1.563 estudos registrados, sendo 366 de fases III e IV. Vários tratamentos antivirais e imunomoduladores estão sendo testados em vários estágios da COVID-19 e serão publicados nos próximos meses. Até que uma vacina esteja disponível, precisamos cada vez mais compreender o que leva alguns pacientes a evoluírem tão mal para, assim, evitar o desfecho fatal através de medidas e tratamentos disponíveis.

Diante da necessidade urgente de se encontrar tratamentos eficazes para a COVID-19 por meio de estudos controlados e randomizados, certos agentes estão sendo usados em diferentes países com base em evidências *in vitro.* Também estão sendo realizadas extrapolações de resultados em outras doenças virais ou ainda sendo feitos tratamentos baseados em estudos observacionais e pequenos ensaios clínicos. Na Figura 2, organizamos as estratégias de tratamento de acordo com a fase da doença.

**Figura 2:** Estratégias de tratamento da COVID-19 de acordo com a fase clínica da doença. *Estudos em andamento; ^†^Ajuste da dose pelo peso e clearance de creatinina (ClCr); ^‡^ECMO (circulação por membrana extracorpórea); ^§^Quando forte suspeita clínica de tromboembolismo venosos ou confirmação de embolia pulmonar ou trombose venosa profunda proximal (ajuste ao peso e ClCr).

Entre os fatores de risco relacionados à evolução desfavorável da doença, podemos dividi-los em não modificáveis e modificáveis. Sexo masculino e idade avançada são fatores não modificáveis. Entretanto, as pessoas mais idosas devem ser prioritariamente isoladas, principalmente se apresentam doenças inflamatórias crônicas^25^. Os tabagistas ativos também inspiram mais cuidado, visto que o pulmão é o órgão onde se iniciam as complicações da doença a partir da fase II, além de ser um importante fator de risco para aterosclerose^1^.

Os pacientes com DCV, síndrome metabólica e seus principais componentes (obesidade, diabetes e hipertensão) merecem muita atenção^24^. O controle terapêutico desses fatores parece uma medida fundamental como forma de deixar o endotélio menos reativo e, assim, menos vulnerável à COVID-19^40^. A otimização do tratamento medicamentoso com o uso de hipoglicemiantes, anti-hipertensivos, hipolipemiantes (principalmente as estatinas) e antiagregantes plaquetários [como o ácido acetil salicílico (AAS)] poderia ajudar a estabilizar o endotélio na tentativa de deixá-lo menos reativo.

As drogas como IECA e BRA ajudam a equilibrar o SRAA. Como discutido anteriormente, esse sistema parece estar mais desregulado na COVID-19. Além do mais, estudos observacionais em pacientes hospitalizados com COVID-19 têm sugerido menor risco de morte nos pacientes em uso dessas medicações, especialmente IECA^18^.

Uma vez infectado com o SARS-CoV-2, o primeiro passo seria o uso precoce de drogas que possam impedir a replicação e favorecer o clareamento viral. Ainda não temos a droga ideal, mas estão em andamento no mundo inteiro estudos com antimaláricos, antivirais, antibacterianos e antiparasitários. Há relatos não publicados de sucesso com a abordagem de *hit early and hit hard* (ataque precocemente e ataque intensamente). Essa hipótese está sendo testada em ensaios clínicos multicêntricos em âmbito nacional e em outros países com algumas dessas classes de drogas^56^^,^^57^.

Diante de uma pandemia, as condutas vão sendo readequadas de acordo com a evolução do conhecimento e muito do que já é rotina no tratamento de pacientes graves está sendo aplicado no tratamento da COVID-19. As principais complicações da COVID-19 são SARS, SIC, TEV e CIVD. Todas elas são bem conhecidas na sepse e na COVID-19 parecem ser consequência da resposta inflamatória desregulada.

A SARS seria a primeira grande complicação e existem protocolos específicos de tratamento^4^^,^^7^. Medidas como suplementação de oxigênio, suporte em UTI e ventilação mecânica são fundamentais.

Ainda na fase hiperinflamatória, as drogas que inibam ou reduzam os efeitos das citocinas pró-inflamatórias parecem muito pertinentes. Os inibidores de IL-6, assim como glicocorticoides, poderiam evitar ou amenizar a tempestade de citocinas. Novas medicações moduladoras da resposta inflamatória são fundamentais nessa fase para evitar a inflamação excessiva, que agride intensamente o endotélio e diversos órgãos, podendo culminar com falência de múltiplos órgãos até a morte^58^.

Estudos mostram maior risco de trombose venosa profunda (TVP) e EP em pacientes em pacientes hospitalizados com COVID-19^49^, sendo importante acompanhar esses pacientes com escore clínico para TVP e EP^59^. Em relação ao TEV, os estudos indicam que pacientes hospitalizados devem receber tromboprofilaxia farmacológica com HBPM ou fondaparinux (preferencialmente com HNF), a menos que o risco de sangramento exceda o risco de trombose^31^^,^^47^^,^^51^.

O ajuste de dose da heparina para obesidade e para pacientes com insuficiência renal é recomendado (Tabela 1)^25^^,^^51^. Não temos ainda estudos que possam embasar o uso de anticoagulantes em pacientes sem necessidade clínica de hospitalização pela COVID-19, bem como o uso de doses intermediárias de anticoagulação, sem que ao menos exista uma forte suspeição de TEV. Pesquisas estão em andamento para o uso de anticoagulantes nas diferentes fases da COVID-19. A sugestão da manutenção da profilaxia após a alta hospitalar é uma tendência devido ao estado de hipercoagulação desencadeado pela doença^50^^,^^60^^-^62.

**Tabela 1:** Sumário adaptado do consenso de recomendações de terapia antitrombótica durante a pandemia da COVID-19.

**Fases da COVID-19**	**Terapia antitrombótica**
AMBULATORIAIS	· Estimular deambulação;
**FASE I**	· Avaliar risco de TEV *versus* risco hemorrágico;
	· Considerar profilaxia farmacológica se alto risco trombótico SEM alto risco hemorrágico;
	· Pacientes em uso de terapia antitrombótica: manter tratamento;
	· Pacientes usuários de antivitamina K sem controle adequado: sugerir transição para anticoagulante oral direto ou enoxaparina.
HOSPITALIZADOS	· Avaliar risco de TEV *versus* risco hemorrágico;
**FASE II e III SEM CIVD**	· Iniciar HBPM dose profilática - Enoxaparina 40 mg via subcutânea 1 vez ao dia* (se contraindicada, usar profilaxia mecânica).
HOSPITALIZADOS	· Profilaxia farmacológica se risco de sangramento ausente;
**FASE III**	· Sem recomendação de doses intermediárias ou plenas de HBPM ou de HNF de rotina;
**COM CIVD**	· Usuários de anticoagulação plena: manter tratamento. Considerar redução de dose de acordo com risco de sangramento;
	· Usuários de dupla antiagregação plaquetária: avaliar risco/benefício individualizado de suspensão ou manutenção. Se plaquetas > 50.000, manter a terapia dupla; plaquetas entre 25.000 e 50.000, deixar um único antiplaquetário; plaquetas < 25.000, suspender antiagregação;
	· Pós-hospitalização: avaliar risco de TEV e considerar profilaxia farmacológica por até 45 dias. Estimular atividade física e deambulação.

* Evitar enoxaparina se clearance de creatinina < 30 mL/min; optar por HNF 5.000 UI 2 ou 3 vezes/dia. CIVD = coagulação intravascular disseminada; HBPM = heparina de baixo peso molecular; HNF = heparina não fracionada; TEV = tromboembolismo venoso.

O risco de sangramento em pacientes anticoagulados existe, sobretudo à medida que a doença agrava. O padrão de CIVD na COVID-19 é inicialmente trombótico, mas pode evoluir para hemorrágico com a progressão da doença. É muito importante o acompanhamento da contagem de plaquetas e do nível sérico de fibrinogênio, como também a aplicação dos escores de risco de sangramento tipo IMPROVE (*International Medical Prevention Registry on Venous Thromboembolism*). Pacientes com escore IMPROVE < 7 apresentam baixo risco de sangramento e devem seguir com a profilaxia farmacológica. Nos casos de escore > 7, existe alto risco de sangramento, sendo indicada a profilaxia mecânica^63^.

Em relação ao tratamento da COVID-19, a estratégia terapêutica tem se baseado no reconhecimento precoce das complicações e no suporte otimizado para aliviar os sintomas. Até o momento, não existe tratamento específico que seja comprovadamente eficaz para a COVID-19. Na Figura 2, seguem as principais recomendações terapêuticas de acordo com as fases clínicas da COVID-19.

## CONCLUSÕES

A imunopatologia da COVID-19 parece compartilhar o mesmo receptor TLR4 das DCV e síndrome metabólica. Talvez a ativação inapropriada desse receptor seja o fator responsável pela resposta imune exacerbada ao SARS-CoV-2 evidenciada em pacientes com a forma grave da doença.

Em resumo, enquanto aguardamos a vacina, talvez o melhor tratamento para a COVID-19 seja aquele que englobe terapias que melhoram as condições cardiovasculares e metabólicas dos pacientes, além de medicações que reduzam a replicação viral, a hiperinflamação e o risco de trombose.
